# Ophthalmology

**Published:** 1878-10

**Authors:** 


### Ophthalmology.

### Incrustations of Lead upon Cornea and Conjunctiva. (*Journ. de Médecine, Nov.* 1877.)

In an article on visual troubles due to lead-poisoning, published in the *Recueil d'Ophthalmologie*, M. Galezowski, insists upon these latter accidents being due almost always to the use of collyria containing salts of lead in solution. These lotions are dangerous whenever the cornea or conjunctiva are ulcerated, the amorphous lead being precipitated upon the entire surface of the ulcer, where it is soon covered by a thin pellicle of epithelium which prevents its expulsion.

Lead deposits in the cornea are of most frequent occurrence, and give rise to the most inconvenience; those of the conjunctiva are less frequent and do not interfere with vision; they are usually the result of insufflations of powdered acetate of lead for ganular conjunctivitis.

Lead spots of the cornea present the form, usually, of chalky-white or pearl-colored opacities with well-defined borders. They are speedily covered with an epithelial pellicle which gives the surface a shining appearance. They are of little importance, except in so far as they occupy the central portion of the transparent membrane, when they require surgical intervention. Desmarres removes the incrustation by scraping with the triangular knife of Baer. M. Galezowski engages the knife flatways under the opacity and passes it between the transparent cornea and the opacity, gradually, until the latter is removed. The operation, though painful, is not difficult. The pellicle is hard enough, usually, to allow it to be removed in one piece, and the wound left is even, shining, and cicatrizes perfectly without leaving a trace, and without a new opacity.

Glaucoma and Increased Ocular Tension.—From a recent summary in the *Dublin Jour. of Med. Sci.* (June, 1878), we take the following:

Papers have been published by Knies and Weber to the effect that the chief factor in this affection is interference with the drainage of the eye. The former insists that the obliteration of the canal of Fontana plays an important rôle; and the latter concludes that in all forms of the disease the drainage channels become narrowed and finally occluded.

Recently Dr. Stilling has experimented by ligaturing the optic nerve. At first this was followed by neuro-paralytic symptoms; after five or ten days there took place enormous increase of tension, anæsthesia of the cornea and increased fluidity of the vitreous.

He would divide the malady into—glaucoma anticum—due to obstruction of the anterior drainage system as well as Fontana's canal; and glaucoma posticum—depending on obstruction in the vascular sheath of the optic nerve.

Mr. Brailey, of London, has found marked and universal changes in the ciliary body, the circular fibers being specially affected or almost completely atrophied. He closes thus: "It is extremely likely that the ciliary muscle has functions besides that of accommodation, and that it may be concerned in regulating the blood supply. This might explain the effect of an atrophied ciliary muscle in passively keeping up an increased tension, notwithstanding that the periphery of the iris has never been united to the cornea.

---

The last words of Velpeau: *Il faut toujours travailler, mes amis.* (My friends, we must ever be working.)

